# Noise and air pollution during Covid-19 lockdown easing around a school sitea)

**DOI:** 10.1121/10.0009323

**Published:** 2022-02-08

**Authors:** Prashant Kumar, Hamid Omidvarborna, Abhijith Kooloth Valappil, Abigail Bristow

**Affiliations:** Global Centre for Clean Air Research (GCARE), Department of Civil and Environmental Engineering, Faculty of Engineering and Physical Sciences, University of Surrey, Guildford GU2 7XH, United Kingdom

## Abstract

During the Covid-19 pandemic and resulting lockdowns, road traffic volumes reduced significantly leading to reduced pollutant concentrations and noise levels. Noise and the air pollution data during the lockdown period and loosening of restrictions through five phases in 2021 are examined for a school site in the United Kingdom. Hourly and daily average noise level as well as the average over each phase, correlations between noise and air pollutants, variations between pollutants, and underlying reasons explaining the temporal variations are explored. Some strong linear correlations were identified between a number of traffic-sourced air pollutants, especially between the differently sized particulates PM_1_, PM_2.5_, and PM_10_ (0.70 < r <0.98) in all phases and an expected inverse correlation between nitrogen dioxide (NO_2_) and ground-level ozone (O_3_) (–0.68 < r < –0.78) as NO_2_ is a precursor of O_3_. Noise levels exhibit a weak correlation with the measured air pollutants and moderate correlation with meteorological factors, including wind direction, temperature, and relative humidity. There was a consistent and significant increase in noise levels (p < 0.01) of up to 3 dB with initial easing, and this was maintained through the remaining phases.

## I. INTRODUCTION

>Road traffic is the main source of air and noise pollution in urban environments causing negative impacts on human health, especially on children in school environments (Khan *et al.*, 2018). Many schools are located near busy roads providing access to school children, teachers, staff, and parents. Queues of idling cars during drop-off and pick-up hours emit disproportionally higher pollutants adjacent to schools, exposing the school children to air and noise pollution (Ibrahim and Richard, 2000; Kumar *et al.*, 2020a). As a chronic stressor, noise has the potential to disrupt executive functioning in school children, which includes working memory, decision making, and self-regulation of emotions/behaviours (Belojevic *et al.*, 2012). According to the European Environment Agency (2020), approximately 82 million people living in urban areas in Europe are exposed to noise pollution (Lden ≥ 55 dB). Hänninen *et al.* (2014) found transportation noise (along with secondhand smoke and radon) second only to air pollution with respect to the environmental burden of disease in six countries (Belgium, Finland, France, Germany, Italy, and the Netherlands). Their study included only severe sleep disturbance (with a fairly low onset threshold of 35 dB), and heart disease from road traffic noise, the annoyance which has by far the largest and most widespread impact according to the World Health Organization (2018), was excluded. As transportation is a source of both air and noise pollution, research has explored correlations between air and noise pollution sourced from traffic. This has been done for two key reasons: first, identifying the variability in correlations between air and noise pollution is essential to understand their impacts on human health, and second, to explore the potential of proxy measures for the more difficult to measure pollutants. Examples include Davies *et al.* (2009) finding a moderate correlation between noise and NO_2_ (0.53) and NOx (0.64) during a 2-week measurement period, Fecht *et al.* (2016) reporting moderate correlations between modelled noise and air pollution (0.34–0.55), and Khan *et al.* (2020) using an aggregate modelling approach and finding a weak to moderate correlation between noise and air pollutants (0.01–0.42) in two Danish cities. Dekoninck *et al.* (2015), however, find noise to be a good proxy for black carbon. A comprehensive review by Khan *et al.* (2018) showed a substantial variation in air and noise pollution correlation (0.05–0.74) among studies. Although moderate to strong correlations (up to 0.74) have been reported between noise pollution and some of the traffic-related air pollutants, a solid conclusion cannot be drawn. The number of road lanes, vehicles, presence of major intersections, and the spatial unit could influence the degree of correlation and variability in readings (Fecht *et al.*, 2016; Hänninen *et al.*, 2014).

Although traffic is known as one of the main sources of noise pollution for schools in urban areas (Bhang *et al.*, 2018; Clark and Paunovic, 2018; Minichilli *et al.*, 2018; Zijlema *et al.*, 2020), the pandemic and resulting lockdowns in many countries have provided a unique opportunity to observe significant changes in travel behaviour and impacts on noise and air pollution (Bar, 2021; Kumar *et al.*, 2020b; Yang *et al.*, 2021). The significance of this study is to continuously collect noise data on an hourly basis at a school site in a trafficked urban area, which is correlated with the environmental variables and air pollution data. This study aimed to investigate the changes in noise and air pollution levels at a primary school site in Guildford, UK, during 2021 through a series of lockdown eases. We first explored the levels of noise pollution in each phase. Then, we investigated the strength of any correlations between noise and air pollution at the school site during a subsequent easing of restrictions and possible ways forward for noise abatement and control.

## II. METHODOLOGY

### A. Study site

**FIG. 1.**
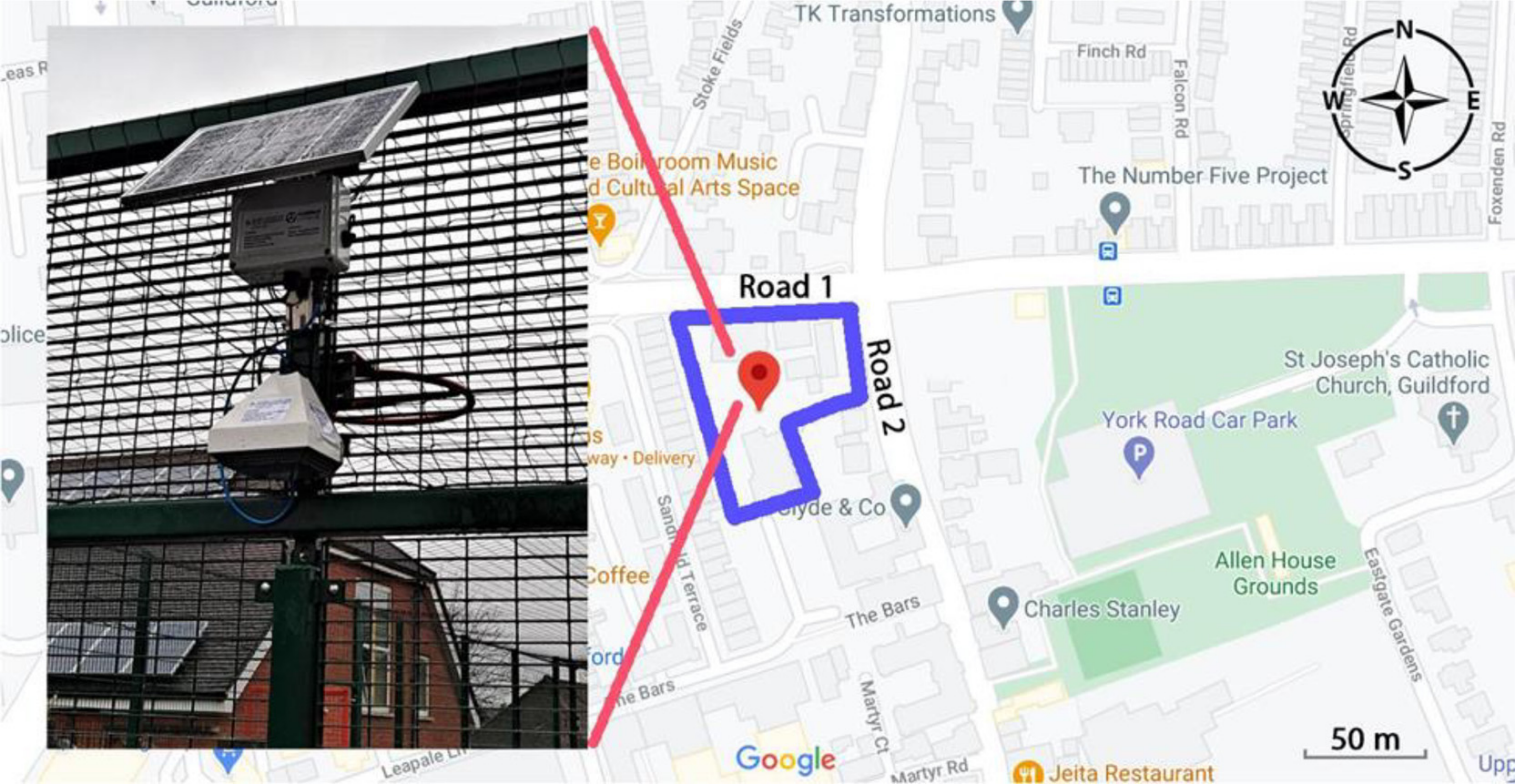
(Color online) The location of the primary school (dark enclosure). The AQMesh pod was installed on a playground cage facing south, approximately 20 metres from road 2 and 30 metres from road 1.

The Sandfield Primary School is located on a heavily trafficked conjunction of two busy roads in Guildford which exacerbates the air quality issues and may create air pollution hotspots at the school premises. This study is a collaboration between the school and Guildford Living Lab (GLL) at the Global Centre for Clean Air Research (GCARE) at the University of Surrey, UK. The collaboration builds upon community engagement and citizen-science activities on air quality with schools in Guildford (see Kumar *et al.*, 2020c; Mahajan *et al.*, 2020). Figure 1 shows the location of the school and the pollution monitor with respect to the roads. The location was determined by the need to identify a site inside the school grounds giving the smallest distance from both roads and the junction, and a requirement to be mounted in a safe place and face towards the South (as it is powered by a fixed solar panel). Apart from the capital and operating cost associated with each unit, performing single point monitoring to gain more understanding about the situation is quite common in research-led studies.

The United Kingdom (UK) was under stringent lockdown measures from January 2021 (beginning of Phase 1). These measures included: (i) stay at home, with limited exceptions; (ii) school closures; and (iii) closure of non-essential shops and services (Prime Minister's Office, 2021). Some schools were open for children of key workers, including this one, but with small numbers of pupils and staff on site. On March 8 (beginning of Phase 2), schools reopened but other measures stayed in place. Subsequent changes on April 12 (beginning of Phase 3) saw the opening of non-essential retail and hospitality venues serving outside and on May 17 (beginning of Phase 4), indoor hospitality and larger outdoor gatherings including some sporting events with spectators. Finally, lockdown measures were removed from July 19 (beginning of Phase 5). Therefore, we defined five phases of lockdown ease: covering January 1 to March 7 (Phase 1); March 8 to April 11 (Phase 2); April 12 to May 16 (Phase 3); May 17 to July 18 (Phase 4); and July 19, 2021 onwards (Phase 5), accordingly.

### B. Instrumentation

The wireless air quality compact system called AQMesh (AQMesh, 2020) is an ambient air quality multi-sensor unit capable of measuring particulate matter (PM) of different size fractions (PM_1_, PM_2.5_, and PM_10_), electrochemical gas sensors [nitrogen dioxide (NO_2_), carbon monoxide (CO), and ozone (O_3_)], air pressure (accuracy of 5 mb), temperature (accuracy of 2 °C), relative humidity (accuracy of 5%), and an omnidirectional microphone for noise measurement (accuracy of 1 dB). For the noise analysis, daily average (L_day_) (7 AM to 7 PM), evening average (L_evening)_ (7 PM to 11 PM), and night average L_night_ (11 PM to 7 AM) of noise level during different phases for weekdays and weekends are defined accordingly. The specification of the main sensors is listed in Table I. The versions of the AQMesh platform for gas protocol and particle protocol were 5.1 and 3.0, respectively. The NO_2_ sensor is designed to reject O_3_ and thus, minimise O_3_-NO_2_ cross sensitivity issues. The recorded raw data were uploaded via a Subscriber Identity Model (SIM) card through General Packet Radio Services (GPRS) communication to a cloud database. While the AQMesh can provide 15 min averaged data, a standard averaging period of 1 h during the period of January to October 2021 is reported here to reduce random noise. Hourly and daily average equivalent noise (L_eq,1h_ and L_eq,24h_) were downloaded from this server for data analysis.

**TABLE I.** The specifications of the main sensors used in the monitoring study in Guildford, UK.

Sensor	Type	Range^a^	LOD	Precision^b^	Accuracy^c^
Noise^d^	Omnidirectional mic	35–100 dB	20–20 000 Hz	>0.8	1 dB
PM	Optical particle counter	0–100 000 (PM_1_)	0 *μ*g m^−3^	(PM_1_ and PM_2.5_) >0.9	5 *μ*g m^−3^
		0–150 000 (PM_2.5_)		(PM_10_) > 0.85	
		0–250 000 (PM_10_)			
		All in *μ*g m^−3^			
NO_2_	Electrochemical	0–20 000 ppb	<1 ppb	>0.85	4 ppb
O_3_	Electrochemical	0–20 000 ppb	<1 ppb	>0.9	5 ppb
CO	Electrochemical	0–1 000 000 ppb	<50 ppb	>0.8	20 ppb

^a^ Obtained from the manufacturer's specification datasheet under standard test conditions (20 °C and 80% of RH) in the absence of any interfering gasses.

^b^ Reported after extensive global colocation experiments against the reference.

^c^ Best accuracy without any local scaling and calibration against the reference.

^d^ Average noise is calculated using all noise samples over the period.

The meteorological data were obtained from Royal Horticultural Society Garden Wisley, United Kingdom [National Grid Reference (NGR) = 5062E 1579 N; altitude = 38 m], which is the closest station to Guildford. All datasets were screened for quality control and quality assurance. The deployed AQMesh pod was calibrated by the manufacturer (Environmental Instruments Ltd, Stratford-upon-Avon, UK) prior to setup and left in operation for a minimum of 2 weeks for stabilisation as advised. The AQMesh exported file comes with a status tag; only data points with valid tags were retained here. Additionally, all negative, N/A, and out-of-spec entries (see Table I) were visually removed from the clean file. Although AQMesh is not designed for regulatory purposes, studies have shown its reliable performance in relative terms (Castell *et al.*, 2018; Margaritis *et al.*, 2021; Wahlborg *et al.*, 2021). The cleaned dataset was analysed using the open-source Openair tools (Carslaw and Ropkins, 2012) in the statistical computing software, R (R Development Core Team, 2013).

## III. RESULTS

### A. Noise pollution

**FIG. 2.**
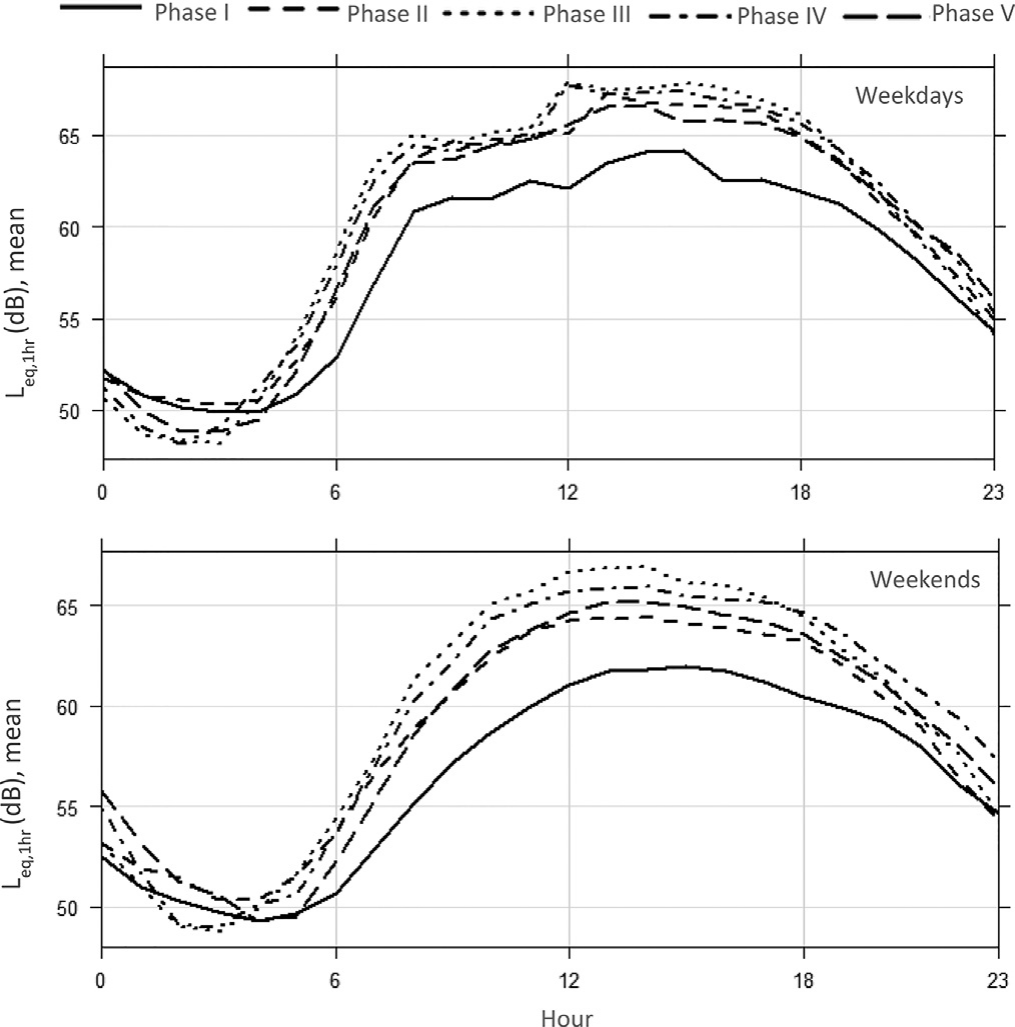
Hourly average equivalent noise (mean L_eq,1h_) during different phases of lockdown eases for weekdays and weekends.

**FIG. 3.**
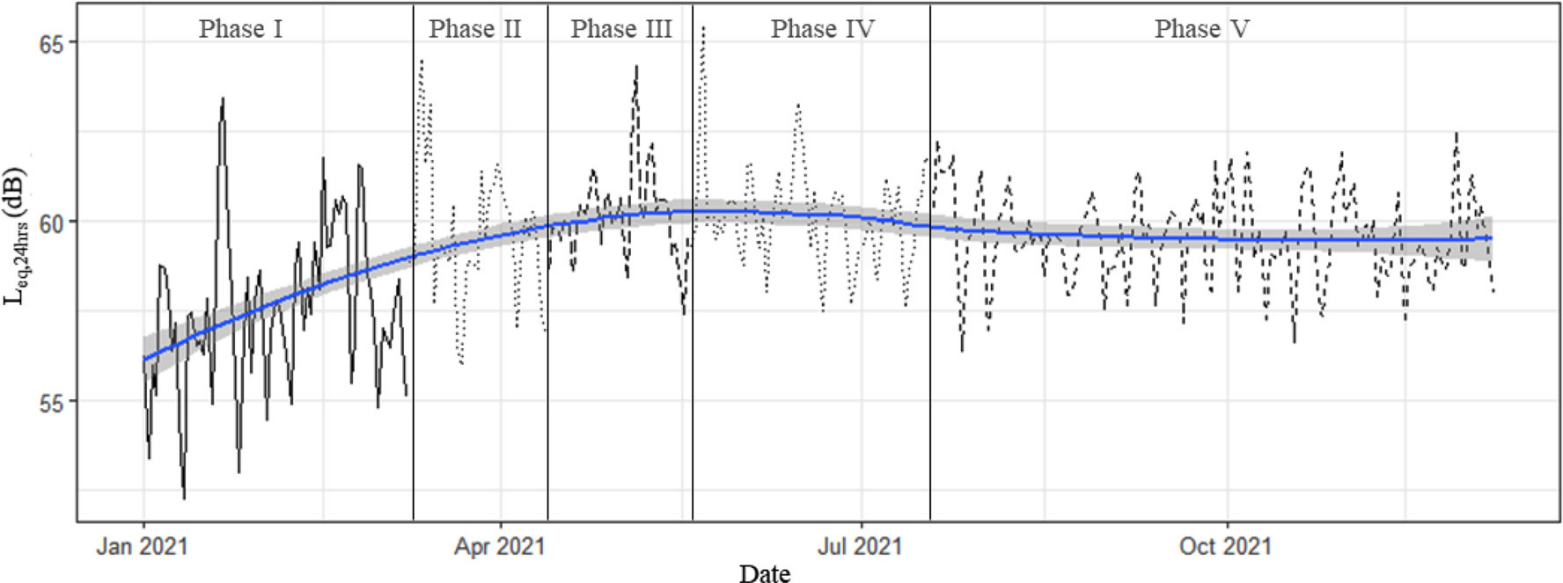
(Color online) Daily average equivalent noise (L_eq,24h_). The line shows a trend and the shaded area around it indicates the 95% confidence interval. The lockdown phases are separated by the vertical lines.

**FIG. 4.**
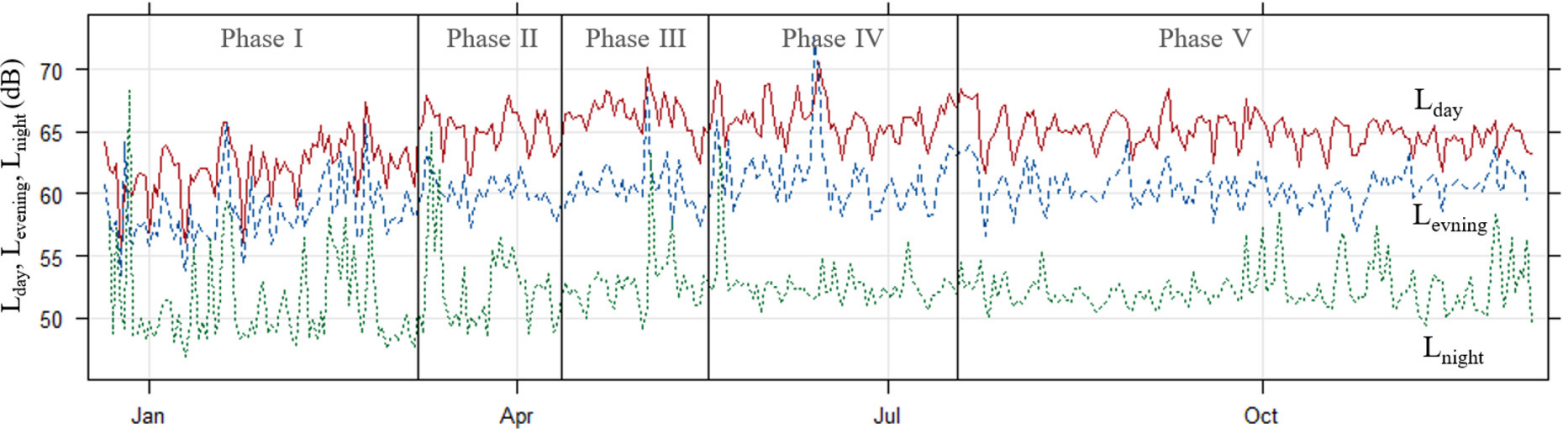
(Color online) Time series plot of average L_day_ (7 AM to 7 PM solid line), L_evening_ (7 PM to 11 PM in dashed line), and L_night_ (11 PM to 7 AM in dashed line) noise level during different phases for weekdays and weekends. The phases are separated by vertical black lines.

**FIG. 5.**
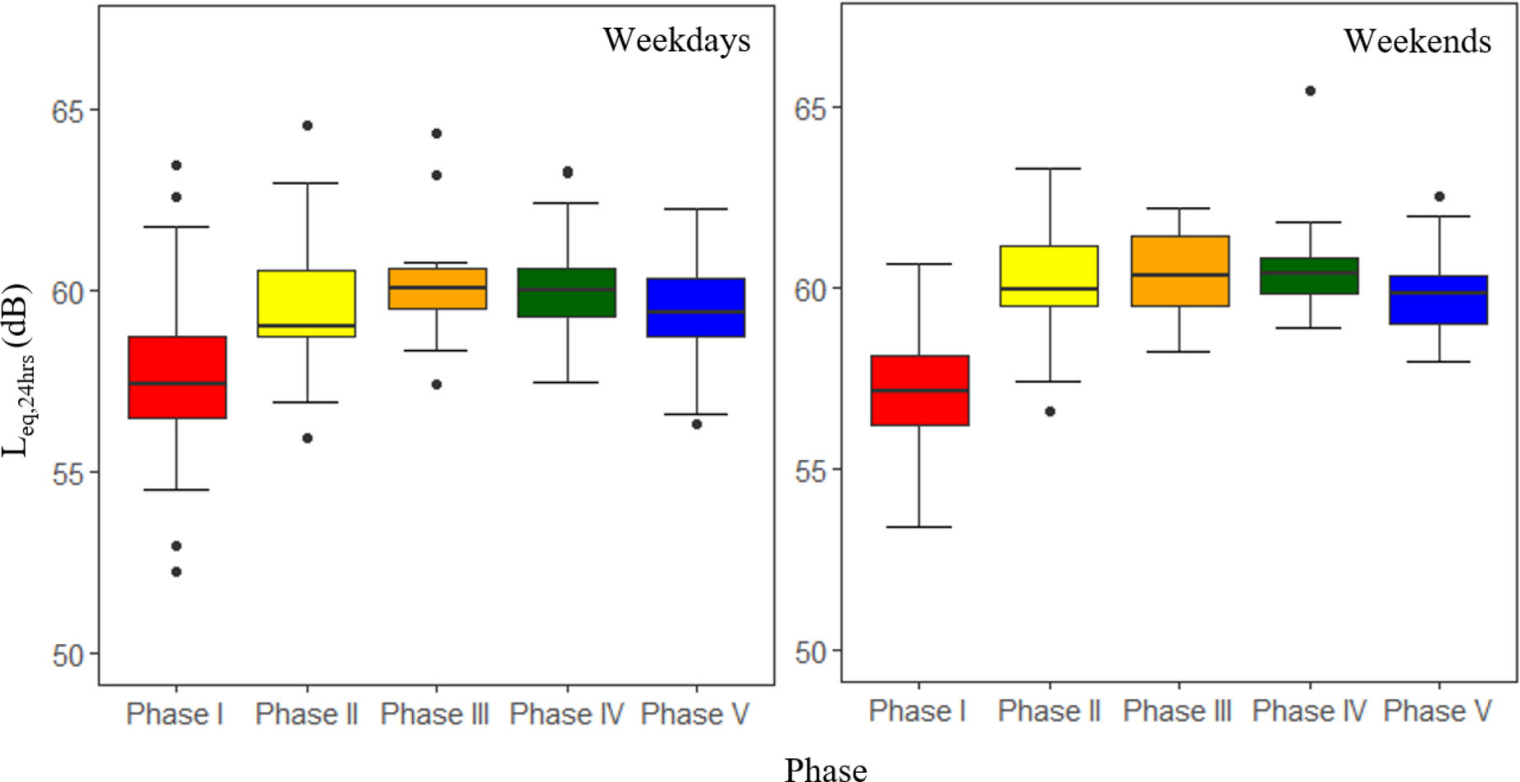
(Color online) Boxplot of average L_eq,24hs_ during different phases at the school site. The lower and upper boundaries of box plots represent the 25th and 75th percentiles, respectively. The lines inside boxes represent median values, while lower and upper error lines represent 1.5*interquartile range below the 3rd quartile and above the 1st quartile, respectively.

Aggregate data for Great Britain shows road traffic levels running at 60%–70% of “normal” levels before March 8 (far higher than in the 2020 first lockdown periods) with goods vehicles at or above normal levels and car use at 50%–60%. After March 8, levels increase to 80% in March and 90% in April approaching normal levels by May and June. It is worth noting that within this car, traffic is running below comparator year levels (90%+) while light and heavy good vehicles are above at around 110% and this continues into October 2021. Interestingly, the largest gap between the comparator year and 2021 is at weekends where traffic levels are consistently higher than they were in a “normal” year; this is most clear for heavier vehicles running 120%–130% of normal—especially on Sundays (Department for Transport, 2021). We, therefore, expect to see some changes in local traffic levels and behaviours, especially at a newly reopened school site. The level of average noise pollution is plotted under different phases of lockdown eases, including L_eq,1h_ (Fig. 2; both on weekdays and weekends), L_eq,24h_ (Fig. 3), daily L_day_, L_evening_, and L_night_ (Fig. 4), and the overall average of each phase (Fig. 5). The data in Fig. 2 seem to support this premise with a small but consistent increase in noise levels especially after Phase 1. On an hourly basis, as shown in Fig. 2, the relative difference started to increase from morning hours around daily commuting time (at around 6:00 am) and continued into the evening (at around 8:00 pm). Figure 2 indicates a similar pattern at weekends, which is in line with the national traffic data. During the lockdown ease period (Phase 2–5), the noise level exceeded by up to 15 dB the recommended average exposure limit of 53 dB L_den_ (World Health Organization, 2018). As our noise measure is not weighted, the exceedance is probably higher. This is a repeated trend, which is also visible in L_eq,24h_ (Fig. 3) as well as L_day_, L_eevening_, and L_night_ (Fig. 4). As shown in Fig. 3, starting from Phase 2, school reopening on March 8, L_eq,24h_ (95% confidence interval) reached to almost the same level as upcoming phases, which indicated that further loosening of restrictions did not make a significant change. The average noise level in the monthly plot (Fig. 3) reveals a steady growth in the fitted line. The average noise levels before and after March 8 were found to be statistically significantly different (*p <* 0.01) using a pairwise t-test. It can be concluded that the school reopening on March 8 was also significant in permitting more freedom of movement for many parents. Despite the comparatively low noise pollution in Phase I, the least variation in L_day_ is detected, starting from the school reopening phase, as shown in Figs. 2 and 4. Notably, the modest difference in L_eq,24h_ during latter phases, see Fig. 5, was extended to weekends, when only <2 dB difference was recorded, as shown in Table II. Such noise pollution from road traffic could result in adverse health effects to both school children and nearby residents.

**TABLE II.** Mean L_eq,24h_, mean L_d_, L_e_, and L_n_ ± Standard Deviation (SD) during different phases of lockdown easing for weekdays and weekends.

Day	Phase	L_eq,24hrs_	L_day_	L_evening_	L_night_
Weekday	Phase I	58.0 ± 5.8	62.8 ± 1.9	58.7 ± 2.3	51.4 ± 3.4
	Phase II	60.1 ± 6.6	65.9 ± 1.0	60.5 ± 1.2	52.7 ± 3.4
	Phase III	60.5 ± 7.3	66.8 ± 1.2	61.2 ± 2.0	53.3 ± 2.2
	Phase IV	60.4 ± 6.9	66.4 ± 1.5	60.8 ± 2.1	52.9 ± 2.0
	Phase V	59.9 ± 6.5	65.9 ± 1.1	60.8 ± 1.2	52.4 ± 1.7
Weekends	Phase I	56.4 ± 5.7	60.6 ± 2.3	58.1 ± 2.8	50.9 ± 5.0
	Phase II	58.5 ± 6.0	63.4 ± 1.5	59.3 ± 1.2	52.3 ± 4.3
	Phase III	59.5 ± 6.7	65.5 ± 1.7	60.1 ± 2.0	51.8 ± 2.5
	Phase IV	59.5 ± 6.4	65.0 ± 1.5	61.5 ± 3.4	51.9 ± 1.0
	Phase V	58.8 ± 5.8	63.8 ± 1.2	60.0 ± 1.8	52.4 ± 1.8

### B. Correlations between pollutants

**FIG. 6.**
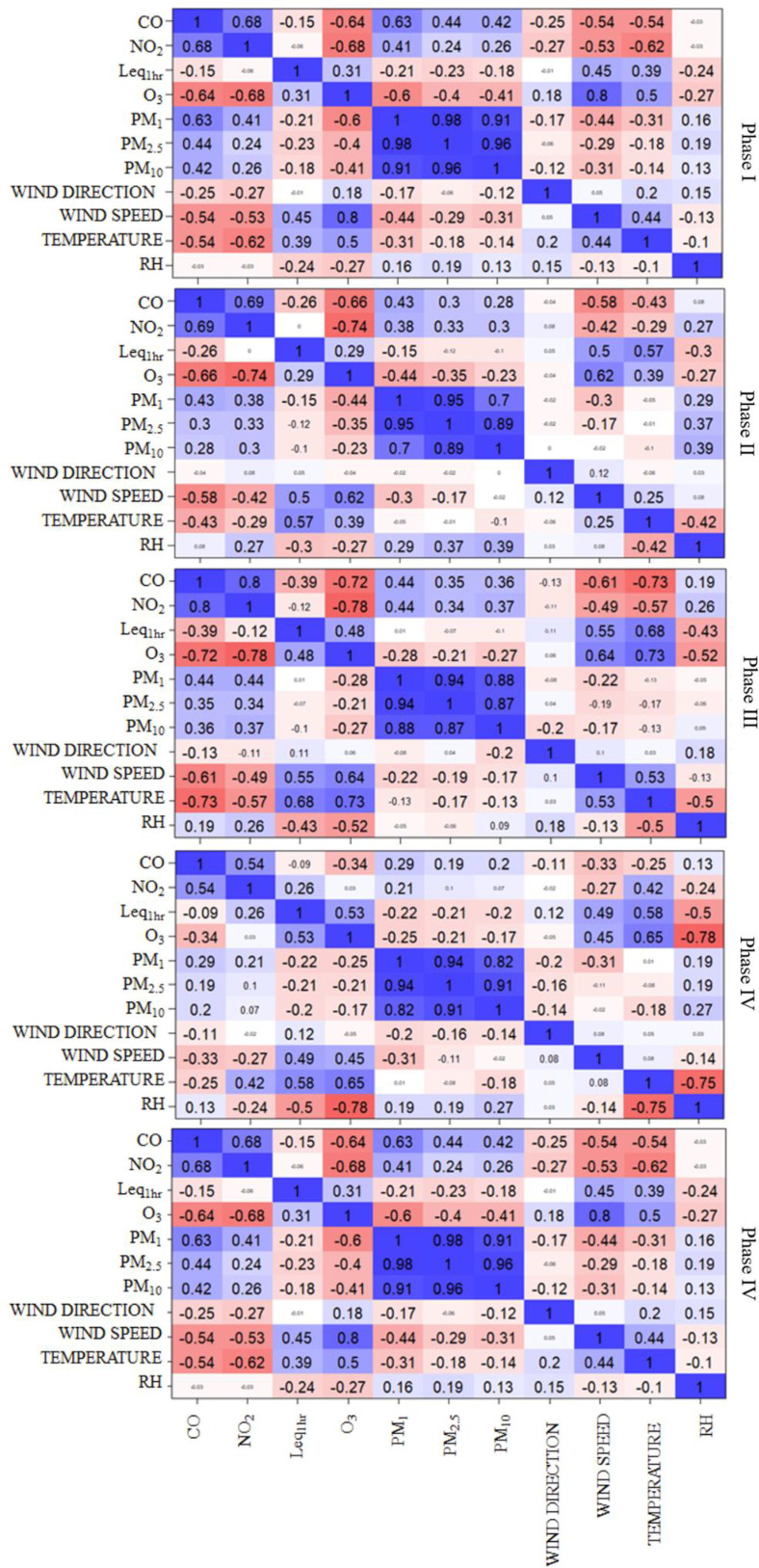
(Color online) Correlation matrix among hourly L_eq,1h_, meteorological parameters and air pollutants during the sampling period (January to end of October 2021) at the school site. The intensity of shading represents the strength of the correlation. Very weak correlations (R < ±0.1) are shown in white and smaller font size.

The Pearson correlation coefficients (r), as plotted for all phases in Fig. 6, show a strong linear correlation between some traffic-sourced pollutants, especially amongst PM_1_, PM_2.5_ and PM_10_ (0.70 < r <0.98) with that between PM_1_ and PM_2.5_ always above 0.90. The inverse NO_2_-O_3_ correlation (r = –0.68 to –0.78) is expected as NO_2_ is one of the precursors of ground-level O_3_. However, there was a time (Phase 4) when no correlation was found between NO_2_-O_3_. Other correlations between air pollutants vary from moderate to weak either positive or negative correlations (r < 0.70), which are shown by moderate/light cool/warm colours and smaller font size. Noise has moderate correlations with wind direction and temperature (positive correlation) and relative humidity (negative correlation), while there is no evident correlation with any of the air pollutants. Moderate to weak positive correlations (0.29 < r < 0.53) between noise and O_3_ were observed. The relatively weak correlations between noise and other air pollutants are probably due to the monitoring station being some distance from the roadside. Further investigations are warranted to also explore the impact of other factors, such as the meteorological parameters (e.g., wind speed and direction) or traffic flow conditions in future studies. For example, Weber and Litschke (2008) also concluded that the homogeneous spatial distribution of noise as compared to the inhomogeneous distribution of PMs could lead to weaker correlations. Correlations between noise and air pollutants have been found to be higher along highways and major roads, roads with multiple lanes, and sampling very close to roads (r = 0.53 between L_eq,5min_ and NO_2_; Davies *et al.*, 2009), which was not the case here.

## IV. CONCLUSION

The study showed a noticeable increase in noise levels in the school site, Guildford, UK, most notably after the March 8 reopening of schools. The data show that the level of noise pollution in the school site exceeds the World Health Organization guideline of 53 dB L_den_. It is likely that the elevated noise pollution is due to an increase in road vehicles after loosening the restrictions. As lockdown eased, noise levels increased by up to 3 dB throughout the week, suggesting the potential for greater noise disturbance at weekends than pre-pandemic. Significant correlations were found between traffic-related air pollutants, especially the different sized particulates. Published literature has reported variations from modest correlation to strong correlations (0.05 and 0.74) between noise pollution and some of the traffic-related air pollutants (Khan *et al.*, 2018). Correlations are usually higher when measured close to the source (i.e., roadside) and weaken with distance. Moreover, unlike noise, the dispersion of air pollutants from the source into the surrounding areas is also driven by atmospheric dispersion conditions, such as wind speed and wind direction. A relatively weaker correlation observed in our case reflects these generic features observed elsewhere as our monitoring was carried out ∼20–30 m away from the source to explain the correlations observed in our case.
